# Anaphylactic Shock Following Multiple Self-Medication Exposures in a Patient With Rheumatoid Arthritis Receiving Methotrexate: A Case Report

**DOI:** 10.7759/cureus.109889

**Published:** 2026-05-29

**Authors:** Mobeen Sajjad, Sammeullah Mirza

**Affiliations:** 1 Department of Medicine, Saad Hospital and Cardiac Care Center, Daska, PAK; 2 Department of Internal Medicine, Saad Hospital and Cardiac Care Center, Daska, PAK

**Keywords:** analgesics, anaphylactic shock, drug interaction, methotrexate, nsaid hypersensitivity, polypharmacy, rheumatoid arthritis

## Abstract

We report a 51-year-old female with rheumatoid arthritis (RA) on long-term methotrexate, hydroxychloroquine, and sulfasalazine, who self-administered multiple medications, including diclofenac 50 mg, without medical supervision. She presented with acute-onset shortness of breath, generalized pruritus, angioedema, and palpitations, and was found to be in severe anaphylaxis with cardiovascular collapse, with a blood pressure of 60/nil mmHg and a heart rate of 143 beats per minute. Diclofenac was considered the most likely trigger based on temporal association and known nonsteroidal anti-inflammatory drug (NSAID) hypersensitivity risk; however, causality cannot be definitively proven as several medications were taken within the same exposure window. Investigations revealed leukocytosis, microcytic anemia, reactive thrombocytosis, and elevated alkaline phosphatase. The electrocardiogram showed no ischemic ST-segment changes or conduction abnormalities. Prompt resuscitation with intramuscular epinephrine resulted in hemodynamic stabilization within 15 minutes of the first dose, with complete angioedema resolution within 30 minutes. The patient was managed without intensive care unit admission and discharged in stable condition on day three. This case highlights the diagnostic and management challenges of severe hypersensitivity reactions occurring in the setting of polypharmacy and emphasizes the importance of individualized analgesic planning and careful medication counseling in patients with RA receiving chronic therapy.

## Introduction

Anaphylaxis is a severe, potentially life-threatening systemic hypersensitivity reaction that demands immediate recognition and intervention. Nonsteroidal anti-inflammatory drugs (NSAIDs) constitute one of the most common drug classes implicated in anaphylactic reactions, with diclofenac, a widely used phenylacetic acid derivative, ranking among the highest-risk agents within this category [1,2]. The pathophysiological mechanism involves IgE-mediated mast cell degranulation as well as non-immunological cyclooxygenase (COX) inhibition, leading to diversion of arachidonic acid metabolism toward leukotriene production and subsequent mast cell activation [3].

Rheumatoid arthritis (RA) is a chronic systemic autoimmune disease characterized by progressive joint destruction and systemic inflammation. Methotrexate, a folate antagonist and cornerstone disease-modifying antirheumatic drug (DMARD) in RA management, is known to modulate immune function through multiple pathways, including adenosine-mediated immunosuppression and inhibition of lymphocyte proliferation [4]. The concurrent administration of NSAIDs with methotrexate carries well-documented pharmacokinetic risks: NSAIDs reduce renal tubular secretion of methotrexate, thereby increasing systemic exposure and toxicity risk [5].

Despite these established risks, patients with RA frequently self-medicate with over-the-counter NSAIDs for breakthrough pain, often without informing their treating physicians. This case report describes a severe anaphylactic reaction with cardiovascular collapse in an RA patient on long-term methotrexate therapy following self-administration of multiple medications, including diclofenac, and underscores the importance of individualized analgesic management in this population.

## Case presentation

A 51-year-old female presented to the Emergency Department of Saad Hospital and Cardiac Care Center, Daska, in the morning hours with a one-hour history of acute-onset shortness of breath, generalized body pruritus, palpitations, and rapidly progressive facial and peripheral angioedema. The patient reported having taken multiple medications at approximately 9:00 AM on the morning of presentation, including diclofenac 50 mg (one tablet), tizanidine 2 mg (one tablet, a centrally acting skeletal muscle relaxant), dexlansoprazole 60 mg, and an antacid suspension (quantity unknown), approximately one hour prior to symptom onset. The preceding night, she had also self-administered tramadol-paracetamol (37.5 mg/325 mg) and dexamethasone 0.5 mg. None of these medications had been prescribed by her current treating physicians.

Her past medical history was significant for rheumatoid arthritis, type 2 diabetes mellitus (DM), and hypertension (HTN). She had no documented history of prior drug allergies or hypersensitivity reactions. No analgesic medications had been formally prescribed by her treating physicians for pain management at the time of presentation. The self-administered medications taken on the day of admission were obtained without a prescription and had not been recommended by her current rheumatologist or any other treating physician. Her complete medication history at the time of admission is detailed in Table 1.

**Table 1:** Complete medication history at the time of admission. BD = twice daily; OD = once daily; TDS = three times daily; MR = modified release; OTC = over the counter; mg = milligrams; Sat/Sun = Saturday/Sunday. * Self-administered without medical prescription. In Pakistan, medications, including tramadol, tizanidine, and dexamethasone, are frequently dispensed without a formal prescription at retail pharmacies, reflecting common dispensing practices in this resource-limited setting. † All four medications were prescribed during neurosurgical follow-up for management of diabetic peripheral neuropathy and associated comorbidities.

Drug	Dose & frequency	Indication	Prescriber
Methotrexate (MTX)	10 mg twice weekly	Rheumatoid arthritis	Rheumatologist
Hydroxychloroquine (HCQ)	200 mg BD	Rheumatoid arthritis	Rheumatologist
Sulfasalazine (SSZ)	500 mg BD	Rheumatoid arthritis	Rheumatologist
Prednisolone	5 mg OD	Rheumatoid arthritis	Rheumatologist
Folic acid	5 mg OD (off Sat/Sun)	MTX supplementation	Rheumatologist
Esomeprazole	40 mg OD	Gastroprotection	Rheumatologist
Carbamazepine	100 mg TDS	Diabetic peripheral neuropathy	Neurosurgeon†
Lacosamide	50 mg BD	Diabetic peripheral neuropathy	Neurosurgeon†
Amlodipine	5 mg OD	Hypertension	Neurosurgeon†
Gliclazide MR	60 mg OD	Type 2 diabetes mellitus	Neurosurgeon†
Diclofenac*	50 mg (morning of admission)	Self-medicated for pain relief	None (OTC)
Tizanidine*	2 mg (morning of admission)	Self-medicated for pain relief	None (OTC)
Dexlansoprazole*	60 mg (morning of admission)	Self-medicated	None (OTC)
Tramadol + paracetamol*	37.5 mg/325 mg (night prior)	Self-medicated for pain relief	None (OTC)
Dexamethasone*	0.5 mg (night prior)	Self-medicated	None (OTC)

On arrival, the patient was visibly distressed with marked angioedema involving the face and bilateral upper extremities. Vital signs on presentation and on ward admission are summarized in Table 2.

**Table 2:** Vital signs at initial assessment (emergency room) and on ward admission. SpO2 = peripheral oxygen saturation; bpm = beats per minute; mmHg = millimeters of mercury; min = minutes.

Parameter	Emergency room	Ward admission	Reference range
Blood pressure (BP)	60/unrecordable mmHg	90/60 mmHg	<120/80 mmHg
Heart rate (HR)	143 bpm	125 bpm	60 to 90 bpm
Respiratory rate (RR)	28 breaths/min	22 breaths/min	12 to 20 breaths/min
Temperature	100°F (37.7°C)	100°F (37.7°C)	97° to 99°F
SpO2	96% (room air)	96% (room air)	>=95%
Glasgow Coma Scale (GCS)	15/15	15/15	15/15

Cardiovascular examination revealed tachycardia with normal heart sounds (S1 + S2, no added sounds). Chest auscultation demonstrated bilateral vesicular breath sounds with no added sounds. Abdominal examination was unremarkable. Cervical lymphadenopathy was absent. A clinical diagnosis of severe anaphylaxis with cardiovascular collapse was established based on established clinical diagnostic criteria for anaphylaxis [6].

Diclofenac was identified as a plausible trigger based on its temporal association with symptom onset and its well-documented association with NSAID hypersensitivity reactions; however, definitive causality could not be established given the multiple concomitant medications administered within the same exposure window.

Resuscitation was initiated immediately with intravenous normal saline 1 L stat, followed by Ringer’s lactate 500 mL, intramuscular epinephrine 0.5 mg (repeated at 15 minutes), intravenous hydrocortisone 100 mg (repeated at 30 minutes), and intravenous pheniramine maleate. The patient demonstrated a rapid and clinically significant response: blood pressure stabilized to 90/60 mmHg within 15 minutes of the first dose of epinephrine, and angioedema completely resolved within 30 minutes. She was subsequently admitted to the general medical ward for monitoring and further management. Urinary catheterization was performed for continuous urine output monitoring in view of hemodynamic instability.

An electrocardiogram (ECG) performed on admission showed no ischemic ST-segment changes or conduction abnormalities (Figure 1).

**Figure 1:**
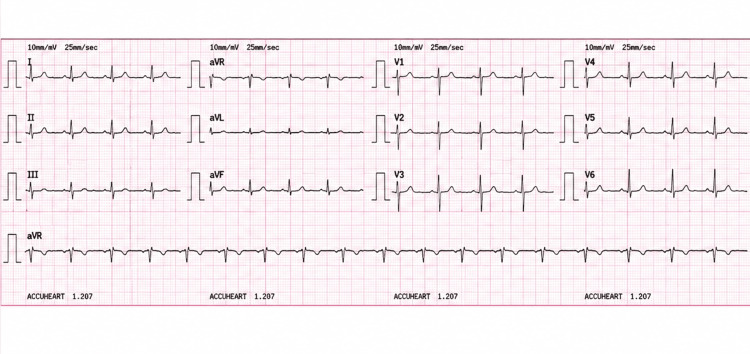
Twelve-lead electrocardiogram performed on admission. The electrocardiogram demonstrated sinus tachycardia without ischemic ST-segment changes or conduction abnormalities. QRS complexes were narrow, and no obvious QTc prolongation was observed.

Investigations

Laboratory investigations performed on admission are detailed in Tables 3, 4. Renal function tests and serum electrolytes were within normal limits.

**Table 3:** Hematological investigations and glycated hemoglobin at admission. fL = femtoliters; pg = picograms; g/dL = grams per deciliter; μL = microliter.

Parameter	Result	Reference range	Interpretation
Glycated hemoglobin (HbA1c)	11.4%	<6.5%	Uncontrolled diabetes mellitus
White blood cell count (WBC)	22,400/μL	4,000 to 11,000/μL	Leukocytosis
Red blood cell count (RBC)	4.02 x 10^6/μL	4.5 to 6.5 x 10^6/μL	Low
Hemoglobin (Hb)	9.3 g/dL	11.5 to 16 g/dL	Anemia
Hematocrit (HCT)	27.0%	38 to 52%	Low
Mean corpuscular volume (MCV)	67.2 fL	80 to 96 fL	Microcytic
Mean corpuscular hemoglobin (MCH)	23.3 pg	27 to 32 pg	Hypochromic
Mean corpuscular hemoglobin concentration (MCHC)	34.6 g/dL	30 to 35 g/dL	Normal
Platelet count	610,000/μL	150,000 to 400,000/μL	Reactive thrombocytosis
Neutrophils	85%	40 to 80%	Neutrophilia
Lymphocytes	10%	20 to 40%	Low
Monocytes	3%	3 to 10%	Normal
Eosinophils	2%	1 to 6%	Normal
Basophils	0%	0 to 8%	Normal

**Table 4:** Biochemical investigations at admission. ALT = alanine aminotransferase; SGPT = serum glutamic pyruvic transaminase; AST = aspartate aminotransferase; SGOT = serum glutamic oxaloacetic transaminase; U/L = units per liter; mg/dL = milligrams per deciliter; Na+ = sodium; K+ = potassium; Cl- = chloride; mmol/L = millimoles per liter.

Parameter	Result	Reference range	Interpretation
Liver function tests			
Bilirubin total	0.9 mg/dL	0.1 to 1.1 mg/dL	Normal
ALT (SGPT)	17 U/L	0 to 38 U/L	Normal
AST (SGOT)	21 U/L	0 to 42 U/L	Normal
Alkaline phosphatase (ALP)	445 U/L	80 to 300 U/L	Elevated, requires follow-up
Renal function tests			
Urea	32 mg/dL	10 to 50 mg/dL	Normal
Creatinine	0.8 mg/dL	0.7 to 1.4 mg/dL	Normal
Blood urea nitrogen (BUN)	15 mg/dL	8 to 22 mg/dL	Normal
Serum electrolytes			
Sodium (Na+)	144.3 mmol/L	136 to 145 mmol/L	Normal
Potassium (K+)	3.77 mmol/L	3.5 to 5.5 mmol/L	Normal
Chloride (Cl-)	108.0 mmol/L	92 to 110 mmol/L	Normal

Inpatient management

The complete emergency and inpatient treatment regimen is summarized in Table 5.

**Table 5:** Emergency and inpatient treatment regimen. IV = intravenous; IM = intramuscular; SC = subcutaneous; BD = twice daily; TDS = three times daily; OD = once daily; PRN = as needed; BP = blood pressure; mL = milliliters; L = liters; hr = hours; mg = milligrams.

Drug/intervention	Dose & route	Frequency	Phase
Emergency department			
Normal saline	1 L IV then Ringer's lactate 500 mL	Stat	Emergency
Epinephrine (adrenaline)	0.5 mg IM	Stat; repeated at 15 min	Emergency
Hydrocortisone	100 mg IV	Stat; repeated at 30 min	Emergency
Pheniramine maleate (Avil)	45.5 mg IV	Stat	Emergency
Inpatient – General Ward			
Normal saline	100 mL/hr IV	Continuous	Inpatient
Hydrocortisone	100 mg IV	BD	Inpatient
Pantoprazole	40 mg IV	OD	Inpatient
Insulin regular (sliding scale)	Sliding scale dose, SC	TDS + PRN	Inpatient
BP and vital monitoring	Not applicable	1 hourly	Inpatient
Foley's catheter + urine output monitoring	Not applicable	Continuous	Inpatient
Discharge - Day 3 (BP = 130/90 mmHg)			
Diclofenac	Discontinued	Not applicable	Discharge
Tramadol	Discontinued	Not applicable	Discharge
Tizanidine	Discontinued	Not applicable	Discharge
Oral antihistamine	Levocetirizine 5 mg PO	OD	Discharge
Prednisolone	20 mg PO	OD (tapering)	Discharge
Glimepiride	2 mg PO	OD	Discharge
Chronic medications	Reviewed and continued as clinically appropriate	Not applicable	Discharge
Biphasic reaction counseling	Documented	Not applicable	Discharge
Analgesic counseling	Future analgesic selection individualized following physician assessment	Not applicable	Discharge

Diclofenac, tramadol, and tizanidine were discontinued at discharge, and the patient was explicitly advised to avoid these medications going forward pending formal allergy evaluation. Sulfasalazine was continued given its chronic use without prior adverse events; however, the patient was counseled regarding the theoretical possibility of sulfasalazine-associated hypersensitivity and advised to report any new symptoms promptly to her rheumatologist. Chronic medications were reviewed and continued as clinically appropriate. An oral antihistamine and a short prednisolone taper were prescribed to mitigate biphasic anaphylaxis risk, which was documented in the patient counseling records. Glimepiride was added given her uncontrolled glycemia (glycated hemoglobin = 11.4%). The patient was discharged in stable condition on day three with a discharge blood pressure of 130/90 mmHg. Future analgesic selection was recommended to be individualized following physician assessment and consideration of allergy evaluation.

## Discussion

This case illustrates a severe anaphylaxis with cardiovascular collapse following unsupervised self-administration of multiple medications in a patient with rheumatoid arthritis on long-term methotrexate, hydroxychloroquine, and sulfasalazine. Several clinically important dimensions merit discussion.

Suspected trigger and pharmacological context

Although diclofenac represented the most plausible trigger based on its temporal association with symptom onset and its well-documented association with NSAID hypersensitivity reactions, definitive attribution was not possible in this case. Multiple medications were taken within the same exposure window, and other concurrently administered agents, including tizanidine, tramadol, and dexamethasone, could not be completely excluded as contributing factors.

NSAIDs may precipitate hypersensitivity reactions through two distinct mechanisms: IgE-mediated mast cell degranulation and non-immunological cyclooxygenase-1 (COX-1) inhibition, the latter leading to leukotriene overproduction and subsequent mast cell activation [3,7]. The patient’s long-term sulfasalazine therapy was considered; however, given its chronic use without prior adverse events and the temporal association of symptoms with the acutely self-administered agents, its role as the primary trigger is considered less likely.

Concurrent methotrexate therapy was clinically noted because NSAIDs are known to reduce renal tubular secretion of methotrexate, thereby increasing systemic exposure and toxicity risk [5]. This pharmacokinetic interaction, however, operates through an entirely different pathway from IgE-mediated or pseudo-allergic hypersensitivity mechanisms. No biochemical evidence of methotrexate toxicity was identified in this case, and its contribution to the anaphylactic event could not be inferred from the available data.

Structured causality assessment using the Naranjo algorithm yielded an overall score of +2, consistent with a possible adverse drug reaction (ADR), reflecting the inherent uncertainty of this polypharmacy context (Table 6) [8]. When applied individually, diclofenac scored +2 (possible ADR), supported by its temporal association and well-documented hypersensitivity profile. Tizanidine scored +1 (possible ADR) based on temporal association alone, with limited published evidence linking it to anaphylactic reactions. These individual assessments further support diclofenac as the most plausible trigger while acknowledging that causality therefore remains uncertain.

**Table 6:** Naranjo adverse drug reaction probability assessment. A total score of +2 indicates a possible adverse drug reaction (ADR), according to the Naranjo scale (score 1 to 4) [8]. Interpretation should remain cautious because multiple medications were administered within the same exposure window.

Naranjo scale question	Score
Are there previous conclusive reports on this reaction?	+1
Did the adverse event appear after the drug was administered?	+2
Did the adverse reaction improve when the drug was discontinued or a specific antagonist was administered?	0 (all medications stopped simultaneously; improvement cannot be attributed to a single agent)
Did the adverse reaction reappear when the drug was re-administered?	0 (not done)
Are there alternative causes that could have caused the reaction?	-1 (polypharmacy; multiple agents taken within the same exposure window)
Did the reaction reappear when a placebo was given?	0 (not applicable)
Was the drug detected in blood in toxic concentrations?	0 (not measured)
Was the reaction more severe when the dose was increased or less severe when the dose was decreased?	0 (not applicable)
Did the patient have a similar reaction to the same or similar drug previously?	0 (no documented prior similar reaction)
Total score	+2 - Possible adverse drug reaction (ADR)

Self-medication, polypharmacy, and clinical risk

A critical contributing factor was the patient’s self-administration of multiple medications without medical supervision. Patients with rheumatoid arthritis frequently experience breakthrough pain that drives them toward over-the-counter medications [9]. In settings where NSAIDs and other analgesics are dispensed without a formal prescription, patients with complex pharmacological backgrounds may unknowingly place themselves at significant risk.

The absence of a prior allergic history does not preclude anaphylaxis. COX-1 inhibition can precipitate reactions on first or subsequent exposures without classical prior sensitization [10]. Clinicians managing patients with rheumatoid arthritis on complex medication regimens should not rely on a negative allergy history as reassurance against hypersensitivity reactions. Individualized analgesic planning and structured medication counseling at every clinical encounter are therefore essential preventive measures in this population.

Hematological and biochemical findings

The leukocytosis (WBC = 22,400/μL) with neutrophilia (85%) most likely represents stress demargination secondary to the acute anaphylactic event and the underlying inflammatory state of RA, rather than a primary infectious etiology [11]. The absence of a documented infection source supports this interpretation.

Reactive thrombocytosis (platelets = 610,000/μL) is a recognized manifestation of chronic inflammatory conditions, including RA, where elevated interleukin-6 (IL-6) drives thrombopoietin-mediated megakaryocyte proliferation [12]. Microcytic, hypochromic anemia (hemoglobin = 9.3 g/dL; mean corpuscular volume = 67.2 fL) may reflect iron deficiency anemia, anemia of chronic inflammation, or a mixed pattern and would require iron studies for further characterization [13].

The elevated alkaline phosphatase (ALP = 445 U/L) with normal transaminases is a notable finding that warrants follow-up, as isolated ALP elevation may represent an early indicator of hepatic, biliary, or bone-related pathology and cannot be attributed to any single etiology without further evaluation, including hepatobiliary imaging [14].

Management and role of early epinephrine

The rapid hemodynamic response observed in this case underscores the paramount importance of prompt intramuscular epinephrine in anaphylaxis management [15]. Blood pressure stabilized to 90/60 mmHg within 15 minutes of the first epinephrine dose, and angioedema completely resolved within 30 minutes. The patient recovered in the emergency room and general medical ward without requiring ICU admission, demonstrating that early epinephrine administration can preclude critical care escalation even in severe anaphylaxis with cardiovascular collapse.

Serum tryptase levels were not obtained due to resource unavailability in our setting. The clinical diagnosis was established on the basis of recognized diagnostic criteria for anaphylaxis [6], encompassing acute onset, skin and mucosal involvement, cardiovascular compromise, and temporally consistent drug exposure.

Allergy and immunology considerations

Given the polypharmacy context and the inability to definitively identify the culprit agent, avoidance of diclofenac and all NSAIDs was advised at discharge pending formal allergy evaluation. Cross-reactivity among non-selective COX-1 inhibitors is recognized, although the pattern and extent of cross-tolerance vary between individuals and reaction phenotypes [7]. This consideration is clinically relevant in patients with rheumatoid arthritis who may require recurrent analgesic exposure.

Paracetamol at standard therapeutic doses represents a reasonable interim analgesic option pending further evaluation. Selective COX-2 inhibitors should not be initiated without prior formal allergy assessment, given the inability to definitively identify the culprit agent. Referral for allergy and immunology follow-up was recommended to guide structured evaluation, including the possibility of supervised drug provocation testing under controlled conditions following complete clinical recovery.

In our resource-limited setting, formal allergy evaluation may not be immediately accessible. Conservative avoidance of suspected triggers, individualized physician-guided analgesic planning, and patient education regarding early recognition of hypersensitivity symptoms represent a pragmatic and safe interim approach.

Limitations

This case carries limitations inherent to its single-patient nature. Definitive identification of the culprit drug was not possible given polypharmacy. Serum tryptase, skin prick testing, and drug provocation testing were not performed. Urinalysis revealed mild pyuria (WBC = 6-8 per high-power field (HPF)), attributed to uncontrolled diabetes mellitus; urine culture was not performed as the clinical picture was consistent with anaphylaxis. Prior medication history was unavailable, precluding assessment of earlier drug exposures. Methotrexate serum concentrations were not measured; therefore, the pharmacokinetic interaction between methotrexate and concurrent medication exposure could not be directly evaluated.

## Conclusions

This case report describes severe anaphylaxis with cardiovascular collapse in a patient with rheumatoid arthritis receiving methotrexate following self-administration of multiple medication agents. Definitive identification of the culprit drug was not possible in the setting of polypharmacy, with diclofenac representing one plausible but unconfirmed trigger. Structured causality assessment using the Naranjo algorithm yielded a score of +2, consistent with a possible adverse drug reaction. No clinical or biochemical evidence of methotrexate toxicity was identified, and methotrexate-related contribution to reaction severity could not be inferred from the available case data. Future analgesic plan should be individualized following physician assessment and consideration of formal allergy evaluation. Prompt intramuscular epinephrine remains the cornerstone of anaphylaxis management and can be life-saving even in settings without intensive care facilities.
